# OLFML2A mediates cell cycle regulation in triple-negative breast cancer via EZH2

**DOI:** 10.3389/fonc.2025.1750297

**Published:** 2026-01-13

**Authors:** Haining Ding, Yian Chen, Qinghong Yu, Wanzhi Jiang, Xiufei Gao

**Affiliations:** The First Affiliated Hospital of Zhejiang Chinese Medical University, Hangzhou, China

**Keywords:** cell cycle, EZH2, OLFML2A, TNBC, triple-negative breast cancer

## Abstract

**Background:**

Triple-negative breast cancer (TNBC) is recognized as one of the most aggressive and prognostically adverse subtypes of breast cancer. The lack of effective therapeutic targets presents substantial challenges, including impediments in early diagnosis, restricted treatment options, and a pronounced tendency for drug resistance. Despite recent advancements in the diagnosis and management of TNBC, the overall survival rate for patients remains suboptimal. Consequently, gaining a more profound understanding of its biological mechanisms and developing novel therapeutic strategies are imperative scientific priorities in this domain.

**Methods:**

OLFML2A was identified as a potential therapeutic target in TNBC through analyses of the Human Protein Atlas and Kaplan-Meier databases. Its functional role was investigated using OLFML2A knockout (KO) and overexpression (OE) models in MDA-MB-231 cells. Effects on proliferation, cell cycle, and apoptosis were assessed by EdU assays, flow cytometry, RT-qPCR, western blotting, and immunofluorescence. Tumor growth and body weight were monitored *in vivo*, and tumor tissues were examined by H&E staining, RT-qPCR, and western blotting. Proteomic profiling integrated with literature mining identified EZH2 as a key downstream candidate. Their interaction was validated by co-immunoprecipitation, and EZH2 expression and localization under different OLFML2A conditions were analyzed. Rescue experiments using the EZH2 inhibitor GSK126 assessed the functional output of the OLFML2A-EZH2 axis via CCK-8, EdU assays.

**Results:**

OLFML2A acts as an oncogenic gene in triple-negative breast cancer. The silencing of OLFML2A markedly reduced the proliferation of MDA-MB-231 cells, induced cell cycle arrest at the G1 phase, and inhibited tumor growth. In contrast, overexpression of OLFML2A reversed these effects. Comprehensive proteomic and molecular biology analyses further indicated that OLFML2A may play a role in cell cycle regulation through the modulation of EZH2.

**Conclusions:**

Our findings suggest that OLFML2A may facilitate cell cycle progression by regulating EZH2, implicating it as a potential therapeutic target for triple-negative breast cancer.

## 1 Introduction

In 2022, female breast cancer emerged as the second most prevalent cancer worldwide, with an estimated 2.3 million new cases, representing 11.6% of all cancer diagnoses. It also ranked as the fourth leading cause of cancer-related mortality globally, accounting for 666,000 deaths, which correspond to 6.9% of all cancer fatalities (1). Research suggests that the pathogenesis of breast cancer is potentially influenced by a variety of factors. The Global Burden of Disease (GBD) 2019 dataset identifies specific behavioral risks—such as tobacco and alcohol consumption, dietary habits, and insufficient physical activity—as well as metabolic risks, including elevated fasting plasma glucose levels and high body mass index, which merit further investigation (2). On a mechanistic level, breast cancer develops from cumulative molecular alterations that disrupt critical cell cycle checkpoints, resulting in abnormal cell proliferation and genomic instability (3). The disruption of cell cycle regulation is a well-established characteristic of cancer, enabling uncontrolled cellular proliferation. Cyclin-dependent kinases (CDKs) play a crucial role in this regulatory framework, and their aberrant activity is associated with the advancement of malignancies, such as breast cancer. Together with their cyclin partners, CDKs form vital regulatory complexes that promote tumor cell proliferation and metastasis (4). As a result, understanding this complex regulatory network and identifying its key components has become a significant focus in current research endeavors.

EZH2 is localized in the nucleus of breast cancer cells and plays a crucial role in modulating the tumor microenvironment and regulating the cell cycle. It has been implicated in the promotion of epithelial-mesenchymal transition (EMT) by facilitating this process. Specifically, EZH2 can repress the transcription of E-cadherin, resulting in reduced intercellular adhesion (5). Numerous studies have elucidated the complex role of EZH2 in the pathogenesis of breast cancer. Biswas et al. (6) identified that EZH2 regulates genes associated with the cell cycle in breast cancer. Chien et al. (7) demonstrated that EZH2 enhances the migration and invasion of triple-negative breast cancer cells by modulating the TIMP2-MMP-2/-9 pathway. Zhang et al. (8) revealed that EZH2 interacts with TGF-β signaling to promote bone metastasis in breast cancer via integrin β1-FAK activation. Additionally, the same research group discovered that EZH2-directed PROTACs concurrently target oncogenic nodes associated with EZH2 and FOXM1, thereby inhibiting breast cancer growth. Moreover, Mao et al. (9) confirmed that the CRISPR/Cas9-mediated knockout of EZH2 reduces the proliferation and migration of triple-negative breast cancer cells. Based on these findings, we propose that EZH2 likely acts as a key driver in the pathogenesis and progression of triple-negative breast cancer by modulating cell cycle progression.

Olfactomedin-like (OLFML) proteins belong to the family of secreted glycoproteins characterized by the presence of an olfactomedin domain, with OLFML2A and OLFML2B being particularly prominent members. These proteins play a vital role in the development and functional organization of neural and retinal tissues (10). Emerging evidence indicates that OLFML2A may contribute to the pathogenesis of various diseases. For instance, Lu et al. demonstrated an association between elevated OLFML2A expression and specific clinical features in acute myeloid leukemia (11). Similarly, Zhao et al. reported a correlation between increased OLFML2A levels and poor prognosis in triple-negative breast cancer (12). Consistent with these observations, our preliminary gene expression microarray analysis has identified OLFML2A as a potential therapeutic target in TNBC cells (13). However, the regulatory mechanisms governing OLFML2A in TNBC remain inadequately understood. This research aims to identify molecular targets that interact with OLFML2A and to elucidate the mechanisms by which it influences the tumor cell cycle. By clarifying the role of OLFML2A in promoting tumor progression, particularly through cell cycle modulation, we seek to identify novel therapeutic targets for TNBC. Our findings have the potential to contribute to the development of more effective and less toxic treatment strategies, ultimately enhancing patient prognosis and quality of life for individuals affected by this aggressive malignancy.

## 2 Methods

### 2.1 Main reagents and kits

Cell Cycle and Apoptosis Analysis Kit (C1052) were purchased from Beyotime Biotech Inc, Annexin V-APC/PI Apoptosis Kit (E-CK-A217) were purchased from Elabscience Biotechnology Co.,Ltd., Click-iT EdU Cell Proliferation Kit with Alexa Fluor 488 (BL915A) were purchased from Labgic Technology Co., Ltd. Antibodies CDK4 (ab199728), CDK6 (ab241554), Cyclin D1 (ab134175), Cyclin D2 (ab308258), Cyclin D3 (ab289546), Rb (ab181616), p-Rb (ab184796), E2F1 (ab317385) were purchased from Abcam. Antibodies EZH2 (5246T) were purchased from Cell Signaling Technology. Antibodies OLFML2A (DF5039) was purchased from Affinity Biosciences Research Center Co., Ltd., Antibodies β-Actin (ET1702-67) were purchased from Hangzhou HUABIO Biotechnology Co., Ltd., TRIzol reagent (AG21101) was used for total RNA extraction and was obtained from Accurate Biology. Rabbit IgG Nanoselector Magnetic beads (025-101-003) were purchased from Chengdu Critical Point Biotechnology Co., Ltd. CCK8 (BS350B) kit were purchased from Labgic Technology Co., Ltd. GSK126 (S7061) were purchased from Selleckchem.

### 2.2 Cell culture

The MDA-MB-231 human breast cancer cell line utilized in this study was procured from Guangzhou Yuanjing Biotechnology Co., Ltd. The cells were cultured in high-glucose Dulbecco’s Modified Eagle Medium (DMEM) obtained from Jinyuan, Shanghai, which was supplemented with 10% fetal bovine serum and 1% penicillin/streptomycin to facilitate cell proliferation. The cell cultures were maintained under standard conditions at 37°C in a humidified atmosphere with 5% CO_2_.

### 2.3 Lentiviral transduction of cells

The MDA-MB-231 triple-negative breast cancer cell line used in this study was obtained from Guangzhou Yuanjing Biotechnology Co., Ltd. To ensure phenotypic specificity, we generated a panel of isogenic cell models via lentiviral transduction using a pre-engineered MDA-MB-231-Cas9 stable cell line (Guangzhou Yuanjing Biotechnology Co., Ltd. Catalog No. YC-D005-Cas9-H) as the parental line. This Cas9-expressing line carries a hygromycin resistance marker based on the YCas-LV002 backbone (non-fluorescent). Using this platform, we established three genetically defined models: (1) an Empty Vector Control (Control-OE), created by transducing cells with the lentiviral backbone vector without the OLFML2A insert to control for transduction and selection artifacts; (2) an OLFML2A Knockout (OLFML2A-KO) model, generated by infecting cells with a lentiviral sgRNA vector (based on the Source Bioscience backbone YKO-001) targeting OLFML2A; and (3) an OLFML2A Overexpression (OLFML2A-OE) model, produced by transducing cells with a lentiviral vector containing the full-length OLFML2A coding sequence.

### 2.4 Flow cytometric analysis of cell cycle

Flow cytometric analysis was performed to assess the cell cycle distribution of MDA-MB-231 cells. The cells were harvested, washed with phosphate-buffered saline (PBS), and subsequently fixed in 70% ethanol at 4°C overnight. Prior to analysis, the fixed cells were stained in the dark at 37°C for 30 minutes with a solution containing propidium iodide (PI) and RNase A. Fluorescence emission was collected after excitation at 488 nm, and the proportion of cells in each phase of the cell cycle was determined based on DNA content.

### 2.5 Flow cytometric analysis of apoptosis

The apoptotic rate of MDA-MB-231 cells was assessed via flow cytometry utilizing dual staining with Annexin V-APC and propidium iodide. In summary, harvested cells underwent washing with phosphate-buffered saline and were subsequently resuspended in 500 μL of 1× binding buffer. The cell suspension was then stained with 5 μL of both Annexin V-APC and PI, followed by a 15-minute incubation at room temperature under dark conditions. Flow cytometric analysis was conducted immediately following the incubation period.

### 2.6 EDU assay

Cell proliferation was assessed utilizing a 5-ethynyl-2′-deoxyuridine (EdU) labeling kit, in accordance with the manufacturer’s instructions. Following cell plating, the EdU incorporation procedure was performed, which encompassed fixation and permeabilization steps. Fluorescence images were subsequently acquired using an EVOS M7000 imaging system (Thermo USA). For the purpose of analysis, the proliferation rate was quantified as the ratio of EdU-positive nuclei to the total number of nuclei counted.

### 2.7 RT-qPCR analysis

Total RNA was isolated utilizing the TRIzol reagent (Accurate Biology, China) following the protocol provided by the manufacturer. Subsequently, the isolated RNA was reverse-transcribed into complementary DNA (cDNA), and quantitative reverse transcription PCR (RT-qPCR) was conducted using SYBR Green (Accurate Biology, China) to assess the transcript levels of CDK4, CDK6, Cyclin D1, Cyclin D2, Cyclin D3, RB, E2F1, and EZH2. The specific sequences of primers used for amplification are presented in **Table 1**.

**Table 1:** The list of primers used in RT-qPCR for mRNA expression.

Gene	Forward Primer	Reverse Primer
CDK4	TGGAAACTCTGAAGCCGACC	AAAGTCAGCATTTCCAGCAGC
CDK6	CTTGCTCCAGTCCAGCTACG	AGGGCAACATCTCTAGGCCA
Cyclin D1	GTGCATCTACACCGACAACTCC	GTTCCACTTGAGCTTGTTCACC
Cyclin D2	TACACCGACAACTCCATCAAGC	TGCCAGGTTCCACTTCAACTT
Cyclin D3	CCTGGGGGCTCTCATGTTTT	CAGCACGGACTACATAGGGG
RB	GAGGACCTGCCTCTCGTCA	TTAACCAAGCTCTCTCTCTGACAT
E2F1	ATGGTGATCAAAGCCCCTCC	AAACATCGATCGGGCCTTGT
EZH2	ACAGTTCGTGCCCTTGTGTGATA	CACACTCTCGGACAGCCAGGTA

### 2.8 Western blotting analysis

Tissue samples were homogenized in ice-cold lysis buffer with protease and phosphatase inhibitors. After centrifugation at 4°C, supernatants were collected for protein quantification. Samples were boiled, resolved on SDS-PAGE gels, and transferred to PVDF membranes. These were blocked for 30 minutes, then probed with primary antibodies overnight at 4°C. After TBST washes, secondary antibodies were applied for 1 hour at room temperature. Immunoreactive bands were visualized using enhanced chemiluminescence and quantified with a digital imaging system.

### 2.9 Immunofluorescence

The study investigated the localization and expression of CDK4, Cyclin D1, p-RB, RB, E2F1, EZH2 and OLFML2A in MDA-MB-231 cells using immunofluorescence techniques. The cultured cells underwent fixation with 4% paraformaldehyde, permeabilization with 0.1% Triton X-100, and blocking with 5% bovine serum albumin (BSA). Subsequently, the fixed samples were incubated with specific primary antibodies at 4°C, and the nuclei were counterstained with DAPI. Imaging was performed using an inverted fluorescence microscope (EVOS M7000, Thermo Fisher Scientific, USA).

### 2.10 CCK8 assay

For the CCK−8 assay, logarithmically growing cells were seeded in 96−well plates and allowed to adhere for 24h. After replacing the medium with fresh medium containing a concentration gradient of GSK126, cells were incubated for an additional 24h. The medium was then aspirated and replaced with 100 μL of fresh medium containing 10% CCK−8 reagent. Following gentle mixing, the plates were incubated for 2 h, and absorbance was read at 450 nm on a microplate reader.

### 2.11 Co-immunoprecipitation

Following cell lysis, a portion of the lysate was reserved as the Input control. The remaining sample was incubated with either an anti-OLFML2A antibody or a control IgG, together with Protein A/G magnetic beads, at 4°C for 2 to 4 hours. The beads were subsequently isolated by centrifugation and washed three to four times with ice-cold lysis buffer. Bound proteins were eluted by boiling the beads for 10 minutes in 2× SDS loading buffer, followed by separation via SDS-PAGE and analysis through Western blotting.

### 2.12 Animals

Following an acclimation period of approximately one week, the mice were randomly allocated into three experimental groups. Cells from the KO, C-OE, and OE lines, each labeled with EGFP, were cultured until reaching the logarithmic growth phase. Subsequently, each BALB/c-nu mouse was administered a subcutaneous injection of 100 μL containing 1×10^7^ cells into the axillary region. Tumor progression and potential metastasis were systematically monitored after the establishment of the xenografts.

### 2.13 H&E staining

After dissection, the mouse tumor samples were fixed in 4% paraformaldehyde and subsequently processed for paraffin embedding. Histological analysis was conducted on tissue sections stained with hematoxylin and eosin (H&E), utilizing a Pannoramic MIDI light microscope for examination.

### 2.14 Proteomic analysis

Cellular proteomic alterations were examined utilizing label-free quantitative proteomics facilitated by data-independent acquisition (DIA) mass spectrometry. Following cell harvesting, lysis was performed, and proteins were alkylated with iodoacetamide (IAM) in the absence of light for one hour at ambient temperature (22 ± 2°C). Subsequently, proteins underwent digestion, desalting, and analysis via liquid chromatography–tandem mass spectrometry (LC-MS/MS) operating in DIA mode. The resulting mass spectrometry data were processed and analyzed through bioinformatic methodologies.

### 2.15 Statistical analysis

The data are expressed as mean ± standard deviation (SD). All statistical analyses were conducted using GraphPad Prism software (version 9.0). For the purpose of group comparisons, a one-way analysis of variance (ANOVA) was utilized. A p-value of less than 0.05 was deemed statistically significant, with specific significance levels indicated as *p < 0.05 and **p < 0.01.

## 3 Results

### 3.1 Clinical relevance of OLFML2A in TNBC

**Figure 1:**
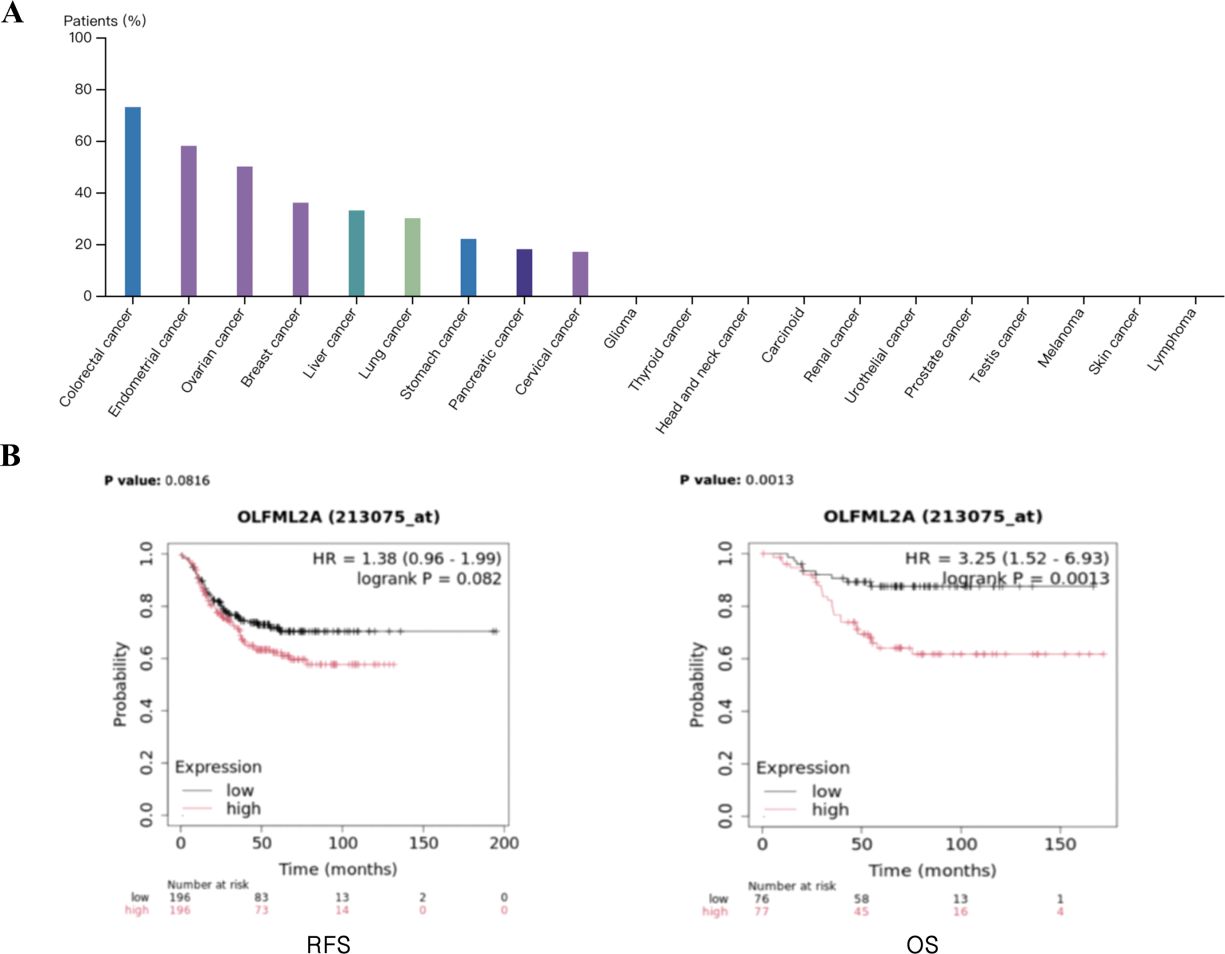
Clinical relevance of OLFML2A in TNBC. **(A)** OLFML2A expression in multiple cancers (Human Protein Atlas). **(B)** Survival analysis of TNBC patients: relapse-free survival (RFS) and overall survival (OS) based on OLFML2A expression (Kaplan–Meier Plotter).

To explore the expression pattern of OLFML2A across various cancer types, we interrogated the Human Protein Atlas database (https://www.proteinatlas.org/). Immunohistochemistry results using the antibody HPA021180 (**Figure 1A**) revealed that OLFML2A exhibits a cancer type-specific expression profile, with notably high cytoplasmic staining in several solid malignancies. Elevated protein levels were consistently observed in colorectal, breast, liver, and pancreatic carcinomas. In contrast, minimal to no OLFML2A expression was detected in cancers such as lymphoma and glioma. This distinct expression pattern suggests a potential tissue- or lineage-specific role for OLFML2A in human oncogenesis. We then evaluated the correlation between OLFML2A expression and the prognosis of TNBC patients using the Kaplan-Meier database (**Figure 1B**). The results showed that high expression of OLFML2A was potentially associated with an increased risk of recurrence in TNBC patients, although this trend did not reach statistical significance (*p* > 0.05). Importantly, high levels of OLFML2A expression were linked to worse overall survival, with the high-expression group having a significantly lower chance of survival compared to the low-expression group (*P* < 0.05). These findings suggest that OLFML2A expression levels could be a potential prognostic indicator for TNBC.

### 3.2 OLFML2A promotes proliferation and suppresses apoptosis in MDA-MB-231 cells

**Figure 2:**
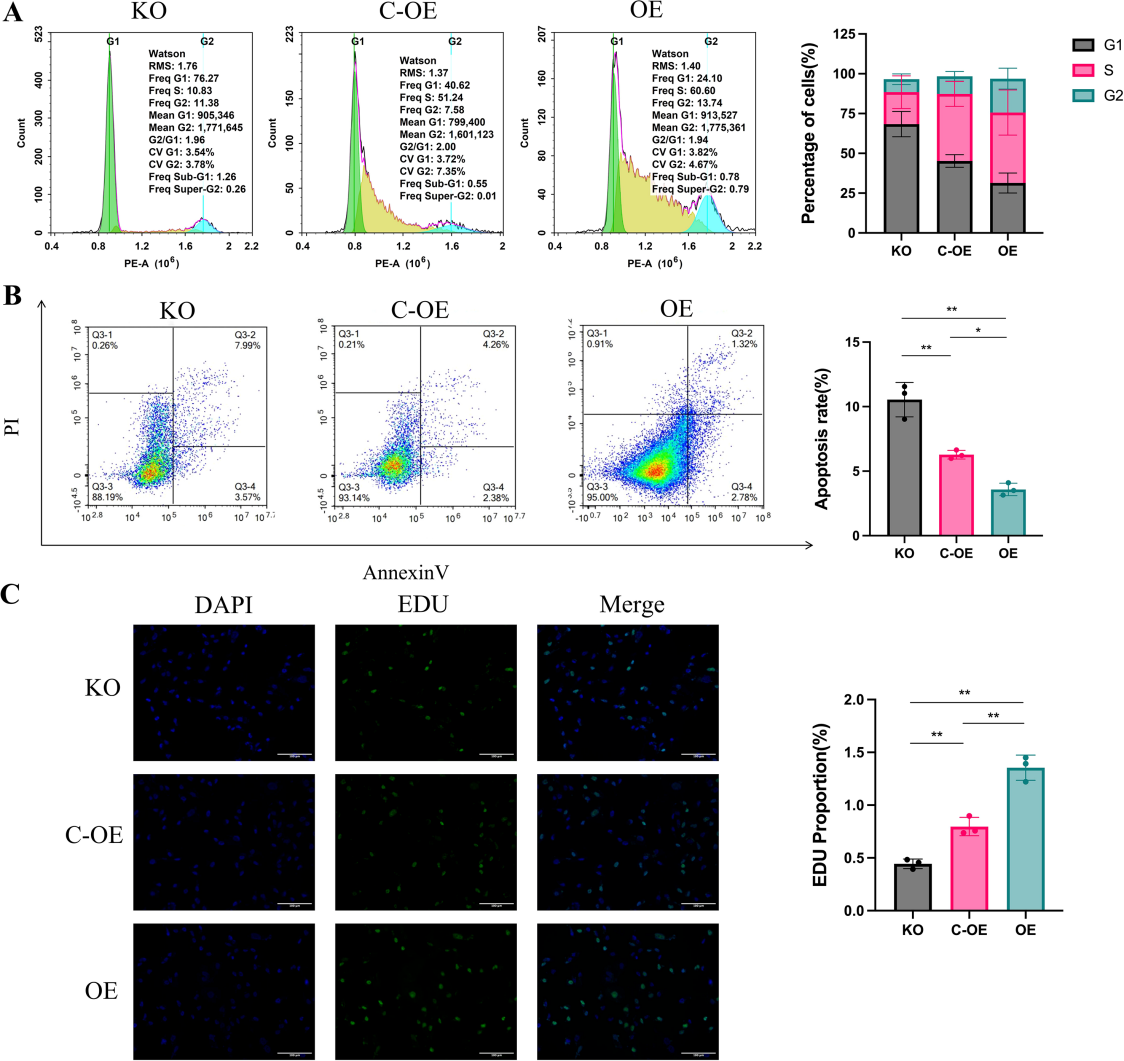
OLFML2A promotes proliferation and suppresses apoptosis in MDA-MB-231 cells. **(A)** Cell cycle analysis by flow cytometry. **(B)** Apoptosis detection by flow cytometry. **(C)** Assessment of cell proliferation capacity via EdU assay. *p < 0.05, **p < 0.01.

To elucidate the biological function of OLFML2A in triple-negative breast cancer cells, we engineered MDA-MB-231 cell line variants with either knockout (KO) or overexpression (OE) of OLFML2A. Flow cytometry was employed to analyze cell cycle distribution and apoptosis. The findings revealed that OLFML2A depletion resulted in a marked accumulation of cells in the G1 phase and an elevated rate of apoptosis. Conversely, OLFML2A overexpression facilitated cell cycle progression and diminished apoptotic activity (**Figures 2A, B**). These results were corroborated by the EdU incorporation assay, which demonstrated that OLFML2A knockout reduced proliferative capacity, whereas OLFML2A overexpression enhanced it relative to control cells (**Figure 2C**). Collectively, these data suggest that OLFML2A plays a critical role in promoting cell proliferation and inhibiting apoptosis in MDA-MB-231 cells, underscoring its potential significance in sustaining tumor cell viability.

### 3.3 OLFML2A induces G1 phase arrest in TNBC

**Figure 3:**
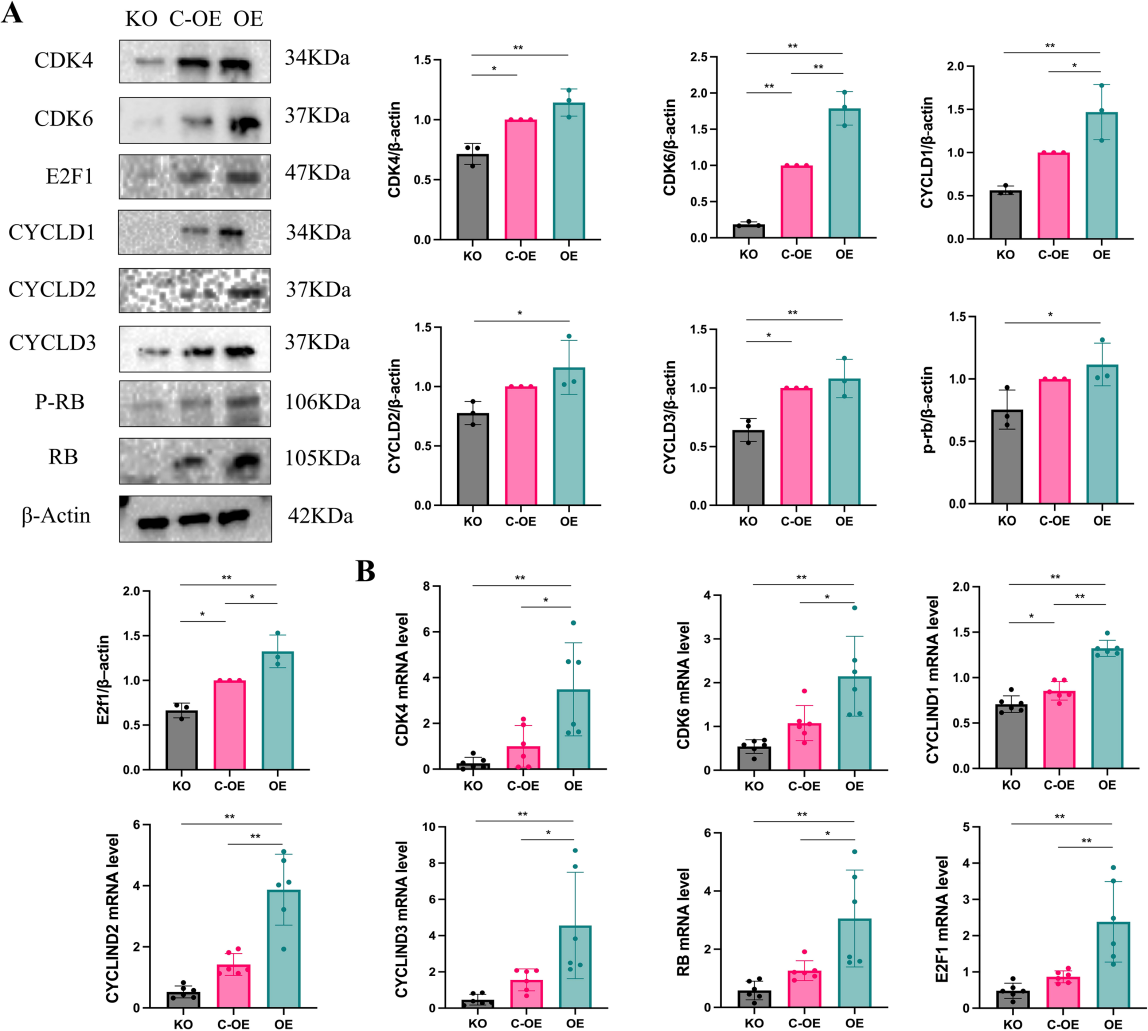
OLFML2A induces G1 phase arrest in MDA-MB-231 cells. **(A)** Representative western blots showing protein expression of G1/S phase transition regulators. **(B)** mRNA expression levels of cell cycle regulatory genes measured by RT-qPCR. *p < 0.05, **p < 0.01.

**Figure 4:**
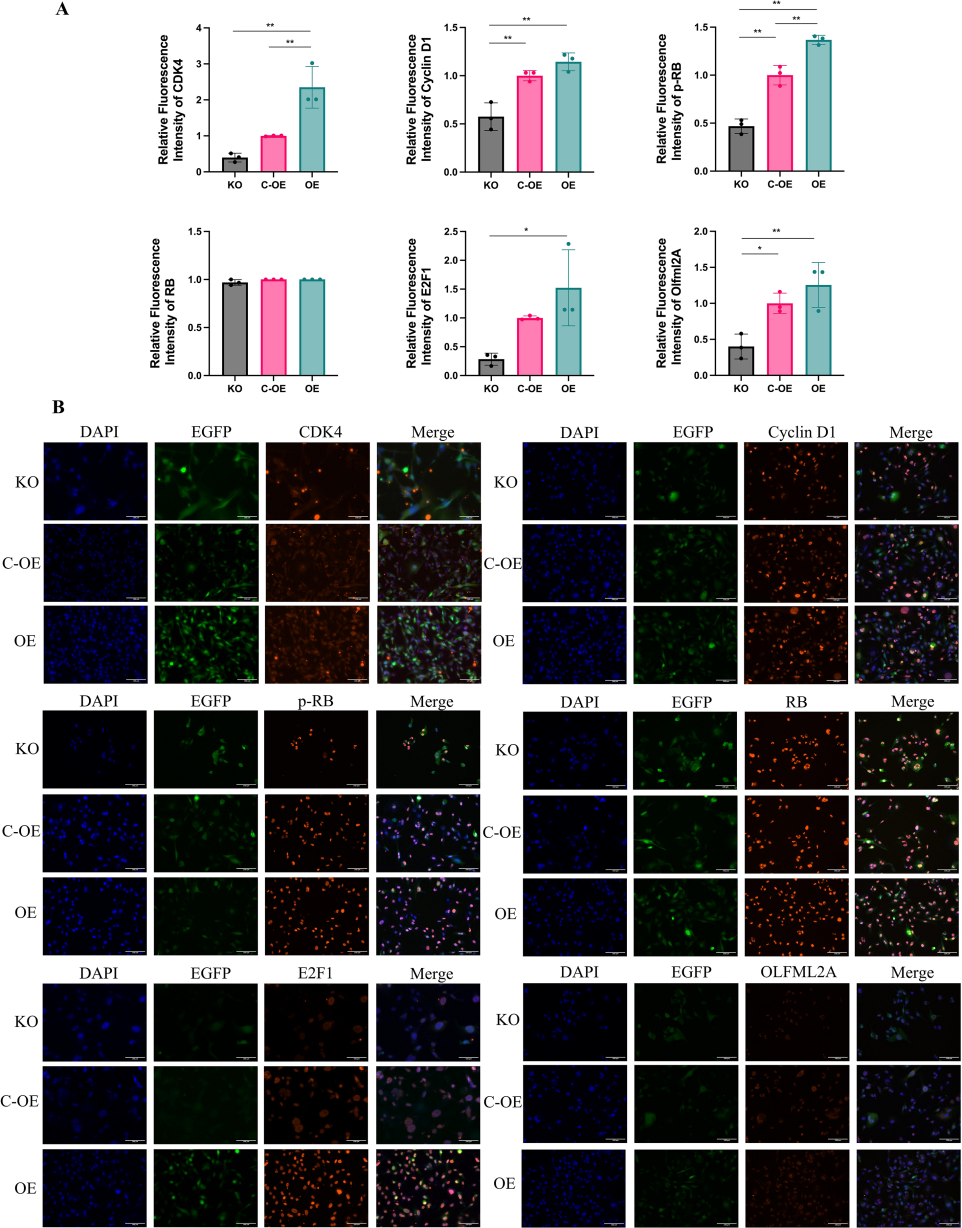
Immunofluorescence analysis of G1 phase-associated biomarkers in MDA-MB-231 cells. **(A)** Quantitative analysis of relative fluorescence intensity for G1 phase regulators. **(B)** Representative immunofluorescence images showing cellular localization of G1 phase markers. *p < 0.05, **p < 0.01.

To elucidate the molecular mechanisms by which OLFML2A induces G1 phase arrest in MDA-MB-231 cells, we conducted an analysis of the expression levels of pivotal regulators involved in the G1/S phase transition. Employing RT-qPCR and western blotting techniques, we evaluated the expression of CDK4, CDK6, Cyclins D1–D3, RB, and E2F1 in both OLFML2A-knockout and control cell lines. The knockout of OLFML2A resulted in a significant reduction in the mRNA and protein levels of CDK4, CDK6, Cyclins D1–D3, and E2F1. Importantly, although the total RB protein expression remained constant, there was a notable decrease in RB phosphorylation (**Figures 3A, B**). These observations were further substantiated by immunofluorescence staining, which visually confirmed the downregulation of these cell-cycle regulators following OLFML2A depletion (**Figures 4A, B**). Taken together, these findings suggest that OLFML2A facilitates G1/S phase progression by modulating the expression and phosphorylation of critical cell-cycle proteins.

**Figure 5:**
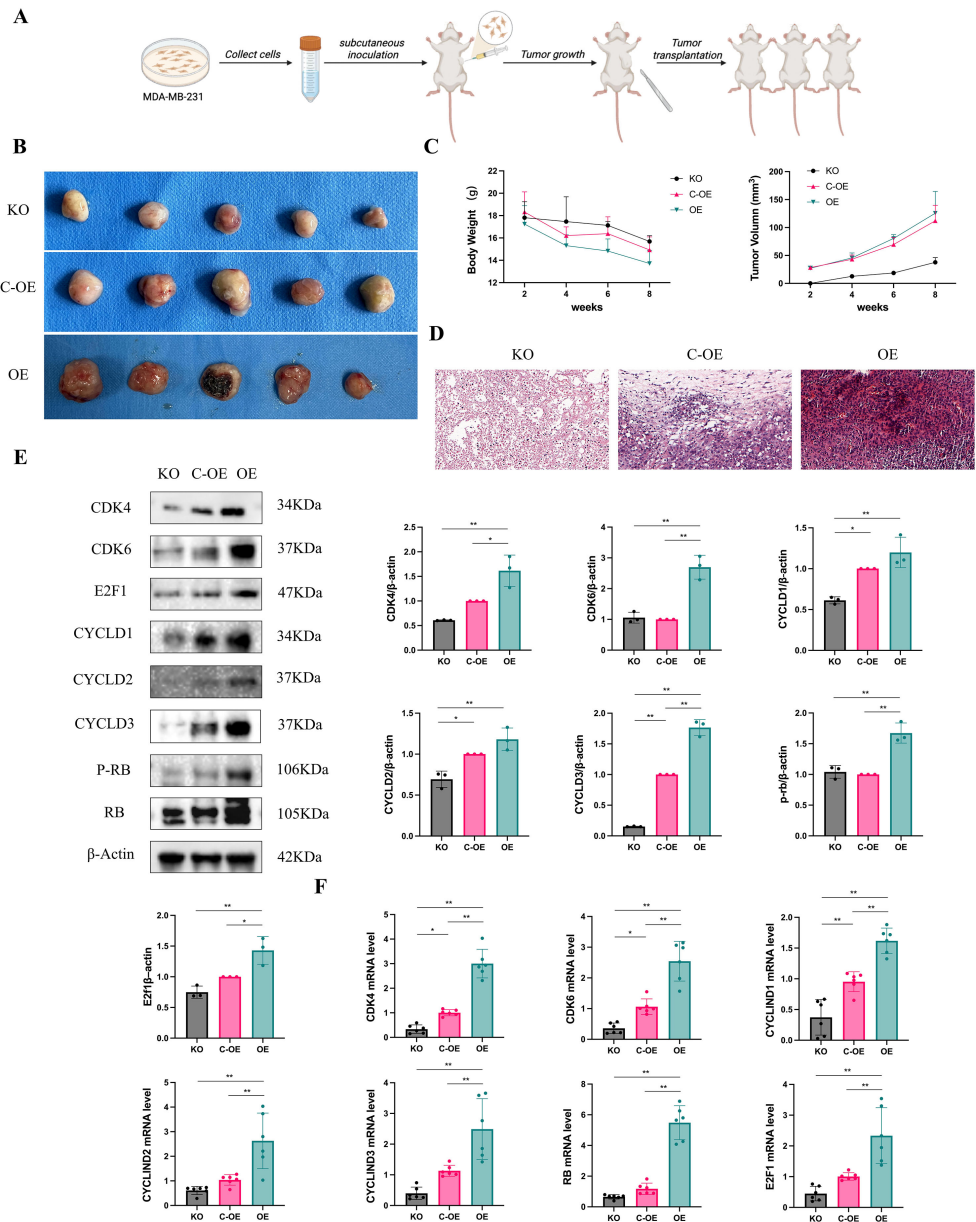
OLFML2A induces G1 phase arrest in a TNBC xenograft mouse model. **(A)** Schematic diagram of the triple-negative breast cancer xenograft model establishment. **(B)** Representative images of excised tumors from each experimental group. **(C)** Body weight and tumor volumn. **(D)** H&E staining of tumor tissues from different treatment groups. **(E)** Protein expression levels of G1 phase-related regulators in tumor tissues. **(F)** mRNA expression of G1 phase-associated markers in tumor tissues. *p < 0.05, **p < 0.01.

To elucidate the role of OLFML2A in the regulation of the tumor cell cycle *in vivo*, we developed a xenograft model of triple-negative breast cancer in mice. All experimental groups exhibited palpable tumors, albeit with distinct growth patterns. Compared to the control group (C-OE), the knockout of OLFML2A resulted in significantly slower tumor growth and reduced final tumor volume, whereas overexpression of OLFML2A led to accelerated tumor progression and increased tumor sizes (**Figures 5A–D**). To investigate the underlying molecular mechanisms, we conducted RT-qPCR and western blot analyses on the tumor tissues. The results indicated that OLFML2A modulates the expression of key regulators of the G1/S transition, including CDK4, CDK6, Cyclin D isoforms, and E2F1, corroborating our previous *in vitro* findings (**Figures 5E, F**). Based on these data, we propose that the depletion of OLFML2A induces G1 phase arrest by inhibiting the CDK4/6–Cyclin D complex, leading to reduced phosphorylation of RB and subsequent suppression of E2F1-mediated transcriptional activation. This pathway likely contributes to the observed inhibition of proliferation and increased apoptosis in breast cancer cells.

### 3.4 OLFML2A interacts with EZH2 to regulate biological processes in TNBC

**Figure 6:**
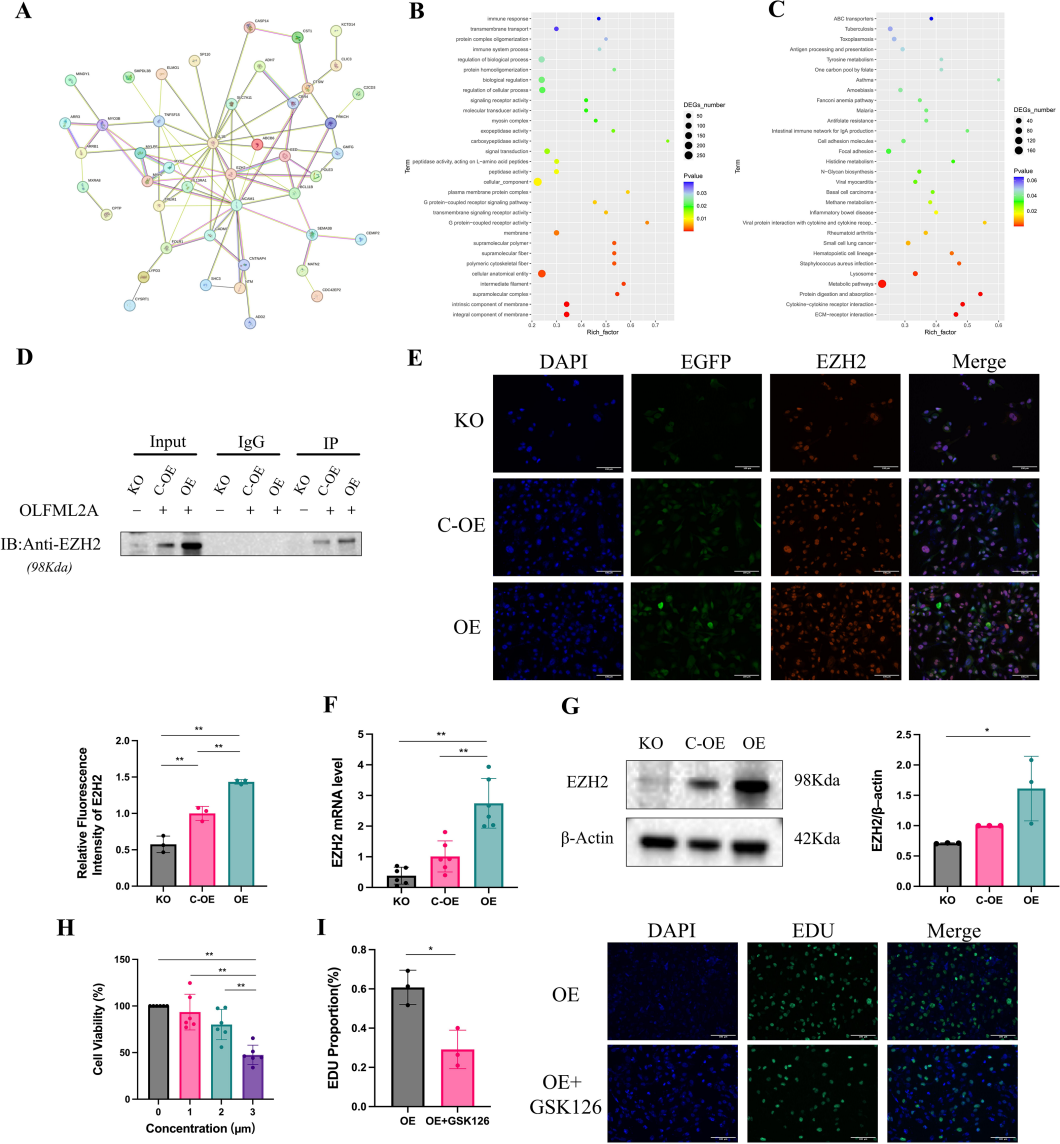
OLFML2A interacts with EZH2 to regulate biological processes in MDA-MB-231 cells. **(A)** Protein-protein interaction (PPI) network and related biological processes revealed by proteomic analysis of KO versus OE groups. **(B)** GO enrichment analysis of the KO versus OE groups. **(C)** KEGG pathway analysis of the KO versus OE groups. **(D)** Validation of EZH2 interaction by co-immunoprecipitation assay *in vitro*. **(E)** Relative fluorescence intensity of EZH2 across experimental groups. **(F)** mRNA expression levels of EZH2 in different cell groups. **(G)** Protein expression of EZH2 among experimental groups. **(H)** CCK−8 assay of OE cell viability following treatment with increasing concentrations of the EZH2 inhibitor GSK126. **(I)** EdU proliferation assay comparing OE cells with OE cells treated with GSK126. *p < 0.05, **p < 0.01.

To elucidate the functional role of OLFML2A in TNBC, we employed the STRING database (https://cn.string-db.org/) to construct a protein–protein interaction (PPI) network. This approach enabled a systematic analysis of potential interaction partners and helped clarify the biological pathways associated with OLFML2A in TNBC. Differentially expressed proteins (DEPs) were identified based on screening criteria of FDR ≤ 0.05, FC ≥ 2, and *p* ≤ 0.05. Comparison between the OE and KO groups revealed a total of 1,383 DEPs, of which 1,026 were up-regulated and 357 were down-regulated. From these, the top 45 proteins ranked by p-value were selected for PPI network analysis (**Figure 6A**). By integrating the proteomic results with literature evidence, we identified EZH2 as one of the key interactors of OLFML2A in TNBC, showing a positive correlation with OLFML2A expression. To elucidate the functional roles of the DEPs, we performed Gene Ontology (GO) enrichment analysis using GOatools (https://github.com/tanghaibao/GOatools) (**Figure 6B**). Enrichment significance was assessed based on Fisher’s exact test, and p-values were adjusted for multiple testing using FDR correction. GO terms with an adjusted p≤ 0.05 were considered significantly enriched. Key enriched biological processes included”immune response”and”immune system process,”while molecular functions such as “signaling receptor activity” and “molecular transducer activity”were highlighted, along with cellular components such as”myosin complex.”Subsequently, Kyoto Encyclopedia of Genes and Genomes (KEGG) pathway enrichment analysis was performed on the DEPs (**Figure 6C**), revealing significant associations with pathways related to immune and infectious diseases, metabolism, cancer and other diseases, as well as cellular structure and signaling.

To validate the interaction between OLFML2A and EZH2, we performed Co-IP assays in MDA-MB-231 cells: KO, C-OE, and OE. Cell lysates were immunoprecipitated using an anti-OLFML2A antibody, followed by immunoblotting with an anti-EZH2 antibody. As shown in **Figure 6D**, EZH2 was readily co-precipitated with OLFML2A in both C-OE and OE cells, whereas the interaction signal was nearly undetectable in KO cells. These results indicate a specific and OLFML2A dependent association between OLFML2A and EZH2 in TNBC cells. Immunofluorescence staining further demonstrated that knockout of OLFML2A significantly diminished EZH2 intensity, whereas overexpression of OLFML2A augmented EZH2 fluorescence signals relative to controls (**Figure 6E**). In alignment with these findings, both mRNA and protein levels of EZH2 were downregulated upon OLFML2A depletion and upregulated following OLFML2A overexpression, as confirmed by RT-qPCR (**Figure 6F**) and western blot analysis (**Figure 6G**). Collectively, these results establish EZH2 as a downstream interacting partner of OLFML2A and suggest that its expression is positively regulated by OLFML2A in triple-negative breast cancer (TNBC) cells.

To investigate the functional role of EZH2 downstream of OLFML2A, we employed the EZH2-specific inhibitor GSK126. First, an appropriate working concentration of GSK126 was determined via CCK-8 assay (**Figure 6H**). Treatment with 3 μM GSK126 resulted in approximately 60% relative viability in OLFML2A-OE cells, indicating effective EZH2 inhibition while preserving basic cellular metabolic activity. At this concentration, EdU proliferation assays were performed (**Figure 6I**). The results demonstrated that GSK126 treatment significantly reduced the percentage of EdU−positive cells compared with untreated OE controls, suggesting that EZH2 inhibition effectively reverses the enhanced proliferative capacity driven by OLFML2A overexpression. Together, these data further support that EZH2 acts downstream of OLFML2A and mediates its pro−proliferative function.

## 4 Discussion

In conclusion, this study elucidates the role of OLFML2A as an upstream regulator that facilitates cell cycle progression in TNBC by enhancing EZH2 expression. The knockdown of OLFML2A resulted in a decrease in both mRNA and protein levels of EZH2, which subsequently induced G1 phase arrest and led to the downregulation of critical cell cycle regulators, including CDK4, CDK6, Cyclins D1–D3, phosphorylated RB, and E2F1. In contrast, overexpression of OLFML2A increased EZH2 expression and promoted the G1/S transition, thereby restoring these regulatory factors. These findings delineate a functional axis in which OLFML2A-mediated upregulation of EZH2 expression supports cell cycle progression, underscoring its potential as a therapeutic target in TNBC.

The initial research team found that microarray analysis revealed OLFML2A-KO significantly altered the expression of 1,140 genes, with 428 genes being upregulated and 712 genes being downregulated. Functional enrichment analysis indicated that these differentially expressed genes are involved in critical biological processes, including DNA synthesis, chromosome alignment, microtubule and cytoskeleton organization, cell motility, cell cycle regulation, and cell necrosis (13). OLFML2A knockdown has been shown to suppress proliferation and induce apoptosis in glioma cells. Mechanistically, OLFML2A downregulation inhibits the Wnt/β-catenin signaling pathway through an increase in amyloid precursor protein (APP) expression and a consequent decrease in stabilized β-catenin. This attenuation of Wnt signaling leads to the reduced expression of key downstream oncogenic targets, including MYC, CD44, and CCND2. The functional significance of this axis was further confirmed *in vivo*, where OLFML2A knockdown suppressed tumor growth in both subcutaneous and intracranial glioma xenograft models by impairing Wnt/β-catenin-dependent proliferatio (14). To investigate the potential molecular mechanisms underlying OLFML2A, we conducted proteomic screening, which led to the identification of EZH2 as an interacting partner. This interaction was subsequently validated through co-immunoprecipitation assays. Further experimental analyses, including quantitative PCR, western blotting, and immunofluorescence, confirmed that EZH2 functions as a downstream effector of OLFML2A. EZH2, known as a catalytic subunit of the Polycomb Repressive Complex 2 (PRC2), facilitates tumorigenesis via both its catalytic and non-catalytic activities. Its overexpression or gain-of-function mutations are closely associated with increased tumor cell proliferation in TNBC (15). These findings indicate that the OLFML2A–EZH2–CDK4 axis may serve as a potential therapeutic target for TNBC.

This study underscores the potential of OLFML2A as a therapeutic target for triple-negative breast cancer; however, several limitations must be acknowledged. Firstly, although we have demonstrated through various experimental approaches that OLFML2A influences tumor cell cycle progression and suppresses apoptosis via the regulation of EZH2, the upstream regulatory networks and detailed molecular mechanisms remain insufficiently understood. Future research should aim to elucidate the precise molecular mechanism through which OLFML2A regulates EZH2. Key questions include whether OLFML2A influences EZH2 ubiquitination, phosphorylation, or protein-protein interactions that affect its stability, which represents an important avenue for further investigation. Secondly, While our *in vitro* and *in vivo* findings support the tumor-promoting roles of OLFML2A and EZH2 in TNBC progression, future large-scale clinical studies are needed to definitively correlate their expression with patient outcomes. Such validation will be essential for fully understanding the biological functions of OLFML2A and for guiding targeted therapeutic development. A critical next step will be to perform direct IHC co-expression analysis of OLFML2A and EZH2 in large, well-annotated TNBC patient cohorts to confirm our findings at the protein level in a clinical context.

Our mechanistic understanding of the OLFML2A−EZH2 axis is primarily based on studies conducted in the representative TNBC model MDA−MB−231. Future research should validate these findings using additional TNBC cell lines that represent distinct molecular subtypes, such as luminal androgen receptor−positive and mesenchymal subtypes, as well as in non−tumorigenic mammary epithelial cells. This expanded validation will help establish the broader relevance of this signaling axis across the heterogeneous spectrum of TNBC and clarify its specificity to malignant progression. Furthermore, studies utilizing orthotopic implantation or metastatic models will be crucial to evaluate the role of OLFML2A−EZH2 signaling in local invasion, distant colonization, and within the native mammary tissue microenvironment. Collectively, such efforts would elucidate the context−dependent functions of OLFML2A and strengthen the preclinical rationale for targeting this axis within a precision oncology framework.

Our study elucidates the molecular mechanism by which OLFML2A facilitates cell cycle progression in TNBC through the transcriptional upregulation of EZH2, thereby promoting the transition from the G1 phase to the S phase. This discovery enhances current understanding in two significant ways: Theoretically, it identifies the OLFML2A–EZH2 axis as a pivotal regulator of the G1 checkpoint within the cell cycle network of TNBC. From a translational standpoint, it not only offers novel mechanistic insights into the aberrant proliferation observed in TNBC but also establishes a robust experimental foundation for the development of targeted therapies aimed at the OLFML2A–EZH2 signaling pathway.

## Data Availability

The raw data supporting the conclusions of this article will be made available by the authors, without undue reservation.
